# Assessing capacity of health facilities to provide routine maternal and newborn care in low-income settings: what proportions are ready to provide good-quality care, and what proportions of women receive it?

**DOI:** 10.1186/s12884-020-02926-8

**Published:** 2020-05-12

**Authors:** Keith Tomlin, Della Berhanu, Meenakshi Gautham, Nasir Umar, Joanna Schellenberg, Deepthi Wickremasinghe, Tanya Marchant

**Affiliations:** 1 Faculty of Epidemiology and Population Health, London School of Hygiene & Tropical Medicine, Keppel Street, London, WC1E 7HT UK; 2 Faculty of Infectious and Tropical Diseases, London School of Hygiene & Tropical Medicine, Keppel Street, London, WC1E 7HT UK; 3 Faculty of Public Health and Policy, London School of Hygiene & Tropical Medicine, 15-17 Tavistock Place, London, WC1H 9SH UK

**Keywords:** Maternal and newborn care, Primary health facilities, Facility delivery, Signal functions, Quality of care, Ethiopia, India, Nigeria, Sub-Saharan Africa

## Abstract

**Background:**

Good quality maternal and newborn care at primary health facilities is essential, but in settings with high maternal and newborn mortality the evidence for the protective effect of facility delivery is inconsistent. We surveyed samples of health facilities in three settings with high maternal mortality to assess their readiness to provide routine maternal and newborn care, and proportions of women using facilities that were ready to offer good quality care. Surveys were conducted in 2012 and 2015 to assess changes over time.

**Methods:**

Surveys were conducted in Ethiopia, the Indian state of Uttar Pradesh and Gombe State in North-Eastern Nigeria. At each facility the staffing, infrastructure and commodities were quantified. These formed components of four “signal functions” that described aspects of routine maternal and newborn care. A facility was considered ready to perform a signal function if all the required components were present. Readiness to perform all four signal functions classed a facility as ready to provide good quality routine care. From facility registers we counted deliveries and calculated the proportions of women delivering in facilities ready to offer good quality routine care.

**Results:**

In Ethiopia the proportion of deliveries in facilities classed as ready to offer good quality routine care rose from 40% (95% confidence interval (CI) 26–57) in 2012 to 43% (95% CI 31–56) in 2015. In Uttar Pradesh these estimates were 4% (95% CI 1–24) in 2012 and 39% (95% CI 25–55) in 2015, while in Nigeria they were 25% (95% CI 6–66) in 2012 and zero in 2015. Improved facility readiness in Ethiopia and Uttar Pradesh arose from increased supplies of commodities, while in Nigeria facility readiness fell due to depleted commodity supplies and fewer Skilled Birth Attendants.

**Conclusions:**

This study quantified the readiness of health facilities to offer good quality routine maternal and newborn care, and may help explain inconsistent outcomes of facility care in some settings. Signal function methodology can provide a rapid and inexpensive measure of such facility readiness. Incorporating data on facility deliveries and repeating the analyses highlighted adjustments that could have greatest impact upon routine maternal and newborn care.

## Background

Maternal mortality – the death of women while pregnant or within 42 days of the end of pregnancy – is estimated to have declined globally by 43% between 1990 and 2015 [1]. Nevertheless, in 2015 an estimated 303,000 women died as a result of complications during pregnancy and childbirth, and the global decline masks substantial regional variation: 88% of all maternal deaths in 2015 occurred in sub-Saharan Africa and Southern Asia [1]. Neonatal mortality – the death of infants within the first 28 days after birth – is also estimated to have declined globally between 1990 and 2017 [2], but 2.5 million newborns are estimated to have died in 2017 and the same regional disparities present themselves, with sub-Saharan Africa and Southern Asia having among the highest neonatal mortality rates [2].

The most widely accepted strategy for reducing maternal and newborn mortality is to encourage women to give birth in a health facility rather than at home [3, 4]. Health facilities which offer maternal and newborn care are more likely to provide infection control and – ideally – are staffed by Skilled Birth Attendants (SBAs). These are doctors, nurses or midwives who are trained to monitor the progress of labour and delivery, to offer basic medical intervention should obstetric complications arise, and to refer women or newborns to more advanced care if this is needed and available. SBAs have been described as the most important intervention in reducing maternal and newborn deaths [5, 6], and increasing the proportion of births at which an SBA is present is a key component of the 2016–2030 Global Strategy for Women’s, Children’s and Adolescents’ Health [7]. In many settings SBAs work exclusively from within health facilities in order to have access to the infrastructure, equipment and medication necessary to provide the most effective care [8].

Between 2000 and 2018 the global proportion of women who gave birth in a health facility is estimated to have risen from 52 to 76% [9] and this increase is thought to have made a significant contribution to the estimated global declines in maternal and neonatal mortality over the same period. However, in settings where the number of maternal deaths per 100,000 live births is high (≥300) [10] the evidence for the protective effect of facility birth is inconsistent [11–15]. This suggests that women in these settings may be attending health facilities but the quality of the service is insufficient to provide the health protection they need. Concern over the poor quality of facility care has also lead to some women avoiding health facilities altogether [8].

Common definitions of the quality of health care have focused upon six core elements: good-quality care should be safe, effective, timely, efficient, equitable and people-centred [16]. Fulfilling these core elements within health facilities is dependent upon a wide range of inputs, and if the quality of care is to be quantified then relevant and consistent measurement of these inputs needs to be applied.

One approach to assessing the quality of maternal and newborn care is the use of “signal functions”. These describe specific actions that should be taken in response to events that can arise during labour and delivery, and were initially devised in relation to obstetric complications. For example, a health facility should respond to a woman suffering a ruptured uterus by performing three signal functions – surgery, a blood transfusion and administration of parenteral antibiotics [17]. Each of these require a minimum set of “components” to be present for the facility to carry it out, in the form of professional expertise, infrastructure, and medical equipment and commodities. To be considered ready to perform a signal function a health facility would need to provide all of the components that it would require.

Signal functions have been developed that correspond to a range of obstetric emergencies [17] and Gabrysh and colleagues have extended this methodology by developing six signal functions that can be applied to routine maternal and newborn care in health facilities [18]. These are 1) the monitoring and management of labour using a partograph; 2) infection prevention for the mother; 3) active management of the third stage of labour; 4) thermal protection of the newborn; 5) immediate and exclusive breastfeeding; and 6) infection prevention for the newborn, including hygienic cord care. Each of these signal functions require a health facility to provide the appropriate staffing, supplies and commodities that are needed to perform it, and a health facility that is ready to perform all the signal functions can be regarded as having the means to offer a good standard of routine maternal and newborn care.

In this study our aim was threefold: we wished to apply signal function methodology to measure the readiness of health facilities to offer good quality routine maternal and newborn care in three settings where maternal/neonatal mortality were high and where facility delivery was being promoted. To do this we took representative samples of health facilities in each setting and conducted cross-sectional surveys to gather data on staffing, infrastructure and commodities that would be analysed as signal function components. We also wished to estimate the proportions of women who delivered in facilities that could be regarded as being ready to provide a good standard of routine care. To do this we used maternity registers at each sampled facility to count the number of women who had delivered in each one in the six months prior to the survey. Finally, we wanted to assess how and why the readiness of facilities to offer good quality routine care could change over time, and so we conducted the surveys twice at two time-points and compared them.

## Methods

### Study settings and data collection methods

This study was conducted within the context of the IDEAS project [19], which aimed to generate evidence to improve maternal and newborn health in Ethiopia, the Indian State of Uttar Pradesh, and Gombe State in North-Eastern Nigeria. In 2008 the Maternal Mortality Ratio (MMR) in Gombe State was estimated by the Ministry of Health to be 1002 [20]; in 2010–12 in Uttar Pradesh it was estimated by the Indian Government to be 292 [21]; while in 2010 the MMR in Ethiopia was estimated to be 523 [22].

Representative samples of public sector primary health facilities were taken from each of these settings in 2012 and 2015. The samples were part of a larger programme of population-level multi-stage cluster surveys for which the designs and sampling strategies have been described in detail elsewhere [19]. In Ethiopia, representative samples of health facilities were taken from 56 districts (Woreda) in the Amhara Region, Oromia Region, Southern Nations, Nationalities and Peoples’ Region and Tigray Region. In Uttar Pradesh, representative samples of facilities were taken from 51 blocks in the six districts of Hardoi, Jhansi, Sultanpur, Maharanjganj, CSM Nagar (renamed Amethi) and Raebareli. In Gombe State, representative samples of health facilities were taken from 10 of the 11 local government areas (excluding Gombe Town). The 2012 surveys were conducted in May/June in Ethiopia and Nigeria, and in November in Uttar Pradesh. The 2015 surveys were conducted in March/April in Ethiopia, May in Nigeria and November in Uttar Pradesh.

At each facility the most senior member of staff was interviewed by a trained member of the survey team. The interview was structured around a standardised questionnaire and responses were entered directly by the interviewer into hand-held electronic devices. Each survey recorded 1) details of staff employed; 2) an inventory, taken by the study interviewer, of infrastructure, equipment and supplies (commodities) that were available and functioning on the day of the survey; and 3) data from facility registers of all the deliveries that had taken place at the facility in the previous 6 months.

### Analytical methods

The facilities included in the analysis were those that would be expected to provide basic obstetric and neonatal care in the community, as indicated by their function in the health service structure in each country. These were health centres (as opposed to health posts) in Ethiopia; primary and community health centres in Uttar Pradesh; and primary health facilities in Nigeria. Skilled Birth Attendants (SBAs) were defined according to country definitions at the time of the surveys and included doctors, registered nurses and assistant nurses/midwives. A facility was considered to have an SBA available if at least one was present at the facility on the day of the survey; this was to reflect the reality for many women in labour who arrive at a health facility unannounced and in need of immediate support.

Of the six routine care signal functions previously described, four were selected that were most dependent upon facility staffing, infrastructure and commodities. These were: management of labour using a partograph; active management of the third stage of labour using prophylactic uterotonics; general infection control for the mother and newborn; and specific infection control for the newborn via clean cord care. Within each signal function a number of practical components were identified - the staffing, infrastructure and equipment/commodities that would be required for it to be performed. The readiness of a facility to perform a signal function was measured by the presence of these components; all the components within a signal function had to be available and functioning at a facility on the day of the survey in order for the facility to be classed as ready to perform that function. If a facility was ready to perform all four signal functions then it was regarded as being ready to offer a good standard of basic maternal and newborn care. The components required to perform each signal function are shown in Table 1.

**Table 1:** Facility components to fulfil four signal functions for basic maternal and newborn care

Signal function	Components
Management of labour using a partograph	SBA present at health facility
	Blood pressure machine
	Foetal stethoscope
	Thermometer
	Blank partographs
	Urine testing kit
	Oxytocin or Ergometrin
Active Management of Third Stage of Labour (prophylactic uterotonics)	SBA present at health facility
	Oxytocin or Ergometrin
General infection prevention	SBA present at health facility
	Source of clean running water
	Soap
	Disposable gloves
	Disinfectant
Newborn infection prevention (clean cord care)	SBA present at health facility
	Sterile cord cutter
	Cord tie

At each sampled facility the number of deliveries which had taken place there in the six months prior to the survey were recorded from the facility registers. These delivery data were then linked to the signal function variables and from this an estimate could be made of the proportion of women who delivered in facilities that were ready to provide good quality basic maternal and newborn care, out of all the deliveries that took place in the sampled facilities.

In each setting a point estimate and confidence interval were calculated for the proportion of facilities that were ready to perform each signal function in 2012 and in 2015. These estimates were compared using chi-squared tests. For each year and setting the median number of deliveries was calculated, and variation between these estimates assessed using a non-parametric k-sample test. To compare the volumes of deliveries in care-ready facilities between 2012 and 2015 the clustering of births within facilities was taken into account and a point estimate and confidence interval calculated for each setting in each year. Data were analysed using Stata 14 software (Statacorp USA).

## Results

In 2012, 166 facilities were surveyed that offered care during labour and delivery: 81 in Ethiopia, 60 in Uttar Pradesh and 25 in Gombe State. Of these, 11 facilities (7%) were excluded from the analysis because of missing data relating to presence of SBAs, availability of commodities or volume of deliveries. In 2015, a total of 305 similar facilities were surveyed: 78 in Ethiopia, 121 in Uttar Pradesh and 106 in Gombe State. Of these, 38 facilities (12%) were excluded from the 2015 analysis due to missing data as described above. The same sampling frames were used in 2012 and 2015, and changes in the sample size between years reflected a change in stakeholders’ priorities.

### Number of deliveries in sampled facilities

In 2012 in Ethiopia, the 76 health facilities with complete data recorded 4439 deliveries in the six months prior to being surveyed, a median of 43 deliveries per facility (Inter-Quartile Range (IQR) 19–78, Table 2). In 2015 a total of 19,278 deliveries were recorded in 78 health facilities in the six months prior to survey, raising the median to 238 per facility (IQR 141–355, *p*-value for difference in medians < 0.001). In 2012 in Uttar Pradesh, 22,235 deliveries in 56 facilities were recorded in the six months prior to the survey, a median of 269 deliveries (IQR 16–695) per facility. In 2015, 38,217 deliveries were recorded in 88 facilities in six months, a median of 289 per facility (IQR 6–797, *p*-value for difference in medians = 0.864). In 2012 in Gombe State, 23 health facilities recorded a total of 1575 deliveries in the six months prior to the survey, with a median of 57 deliveries per facility (IQR 15–90). In 2015, 101 facilities recorded 7154 deliveries, with a median of 39 deliveries per facility in six months (IQR 9–85, *p*-value for difference in medians = 0.644).

**Table 2:** Characteristics of 2012 and 2015 health facility surveys in three study settings

	ETHIOPIA		UTTAR PRADESH, INDIA		GOMBE STATE, NIGERIA	
	2012	2015	2012	2015	2012	2015
Total facilities surveyed	81	78	60	121	25	106
Total with complete data^1^ and analysed	76	78	56	88	23	101
Total deliveries in 6 months prior to survey	4439	19,278	22,235	38,217	1575	7154
Median (IQR) deliveries per facility	43 (19–78)	238 (141–355)	269 (16–695)	289 (6–767)	57 (15–90)	39 (9–85)
p-value for change in deliveries over time^2^		< 0·001		0·864		0·644

1. Complete data on volume of deliveries, availability of commodities or number of staff present

2. nonparametric k-sample test on the equality of medians - X^2^ with continuity correction

### Presence of skilled birth attendants in sampled facilities

At least one SBA was present in 93% of surveyed facilities in Ethiopia in 2012 (95% Confidence Interval (CI) 85–97) and 97% in 2015 (95% CI 90–99, *p* = 0.24. Uttar Pradesh had at least one SBA present in 96% of surveyed facilities in 2012 (95% CI 86–99) and 90% in 2015 (95% CI 81–95, *p* = 0.15). In Gombe State at least one SBA was present in 52% of surveyed facilities in 2012 (95% CI 32–71), falling to 21% in 2015 (95% CI 14–30, *p* = 0.003).

### Readiness to perform four routine-care signal functions in sampled facilities

Tables 3, 4 and 5 show, for Ethiopia, Uttar Pradesh and Gombe State respectively, the 12 commodities that contributed towards the four signal functions, and the proportions of sampled facilities where these were available and operational. The availability of these commodities was then combined with the presence of SBAs to determine the readiness of each facility to perform each routine care signal function.

**Table 3:** Ethiopia: proportions of sampled facilities with SBA and commodities, 2012 & 2015

	2012	2015	
	% (95% CI)	% (95% CI)	P (X^2^)
Present at facility on day of survey			
At least one Skilled Birth Attendant	93 (85–97)	97 (90–99)	0·235
Blood pressure machine	93 (85–97)	99 (91–100)	0·093
Foetal stethoscope	99 (91–100)	100 (−)	0·311
Thermometer	88 (79–94)	95 (87–98)	0·137
Blank partographs	64 (53–74)	79 (69–87)	0·040
Urine testing kit	49 (38–60)	73 (62–82)	0·002
Prophylactic uterotonics	93 (85–97)	96 (89–99)	0·448
Source of clean running water	55 (44–66)	58 (46–68)	0·762
Soap	76 (65–85)	92 (84–97)	0·007
Disposable gloves	95 (87–98)	100 (−)	0·040
Disinfectant	78 (67–86)	94 (85–97)	0·005
Sterile cord cutter	100 (−)	99 (91–100)	0·324
Cord tie	92 (83–96)	100 (−)	0·011
Readiness to perform signal functions			
Management of labour using partograph	32 (22–43)	55 (44–66)	0·004
Prophylactic uterotonics	88 (79–94)	95 (87–98)	0·137
Infection control	43 (33–55)	51 (40–62)	0·332
Clean cord care	85 (76–92)	96 (89–99)	0·024
All signal functions	22 (14–33)	35 (25–46)	0·096

**Table 4:** Uttar Pradesh: proportions of sampled facilities with SBA and commodities, 2012 & 2015

	2012	2015	P (X^2^)
	% (95% CI)	% (95% CI)	
Present at facility on day of survey			
At least one Skilled Birth Attendant	96 (86–99)	90 (81–95)	0·146
Blood pressure machine	98 (88–100)	97 (90–99)	0·566
Foetal stethoscope	52 (39–65)	53 (43–64)	0·850
Thermometer	96 (87–99)	95 (88–98)	0·777
Blank partographs	18 (10–30)	36 (27–47)	0·019
Urine testing kit	38 (26–51)	61 (51–71)	0·006
Prophylactic uterotonics	45 (32–58)	66 (55–75)	0·013
Source of clean running water	84 (72–91)	92 (84–96)	0·134
Soap	96 (87–99)	92 (84–96)	0·293
Disposable gloves	91 (80–96)	89 (80–94)	0·643
Disinfectant	95 (84–98)	91 (83–95)	0·414
Sterile cord cutter	96 (87–99)	86 (77–92)	0·050
Cord tie	77 (64–86)	76 (66–84)	0·929
Readiness to perform signal functions			
Management of labour using partograph	2 (0·2–12)	23 (15–33)	0·001
Prophylactic uterotonics	43 (31–56)	64 (53–73)	0·016
Infection control	73 (60–83)	74 (64–82)	0·932
Clean cord care	71 (58–82)	72 (61–80)	0·983
All signal functions	2 (0·2–12)	23 (15–35)	0·001

**Table 5:** Gombe State: proportions of sampled facilities with SBA and commodities, 2012 & 2015

	2012	2015	P (X^2^)
	% (95% CI)	% (95% CI)	
Present at facility on day of survey			
At least one Skilled Birth Attendant	52 (32–71)	21 (14–30)	0·003
Blood pressure machine	87 (66–96)	92 (85–96)	0·439
Foetal stethoscope	100 (−)	90 (82–95)	0·110
Thermometer	87 (66–96)	72 (63–80)	0·147
Blank partographs	26 (12–48)	7 (3–14)	0·008
Urine testing kit	65 (44–82)	47 (37–56)	0·110
Prophylactic uterotonics	70 (48–85)	73 (64–81)	0·721
Source of clean running water	74 (52–88)	37 (28–47)	0·002
Soap	87 (66–96)	77 (68–84)	0·305
Disposable gloves	83 (61–94)	93 (86–97)	0·115
Disinfectant	91 (71–98)	73 (64–81)	0·069
Sterile cord cutter	87 (66–96)	92 (85–96)	0·439
Cord tie	78 (57–91)	74 (65–82)	0·691
Readiness to perform signal functions			
Management of labour using partograph	17 (7–39)	2 (0.5–8)	0·002
Prophylactic uterotonics	39 (22–60)	17 (11–26)	0·020
Infection control	39 (22–60)	7 (3–14)	< 0·001
Clean cord care	43 (25–64)	15 (9–23)	0·003
All signal functions	17 (7–39)	0	< 0·001

In Ethiopia, the readiness to perform signal functions remained stable or improved between 2012 and 2015. In 2015, over 90% of sampled facilities had the required commodities for clean cord care and prophylactic uterotonics. Approximately half were ready to manage labour using a partograph; this showed the greatest improvement from 2012 when the proportion was 32% (95% CI 22–43). Approximately half of facilities could provide adequate infection control (for those that could not this was mainly due to inadequate supplies of clean running water). Overall the proportion of surveyed primary care facilities in Ethiopia which were ready to perform all four routine care signal functions increased from 22% (95% CI 14–33) in 2012 to 35% (95% CI 25–46) in 2015.

In Uttar Pradesh the availability of commodities also remained stable or improved between 2012 and 2015. In both years approximately three-quarters of surveyed facilities were ready to provide basic infection control and clean cord care. In 2015, only around one quarter were ready to manage labour using a partograph but this had increased substantially from 2012 due to increased availability of blank partographs and urine testing kits. In 2015, two-thirds had prophylactic uterotonics available. Overall, the proportion of primary facilities in Uttar Pradesh which were ready to perform the four routine care signal functions increased from 2% (95% CI 0.2–12) in 2012 to 23% (95% CI 15–35) in 2015.

In Gombe State, the picture was reversed. The availability of most commodities in health facilities fell between 2012 and 2015. The proportion of facilities with a supply of clean running water decreased from 74% (95% CI 52–88) in 2012 to 37% (95% CI 28–47) in 2015. These declines mirrored the fall in the proportion of facilities with an SBA present on the day of the survey and meant that no facilities were ready to perform all four signal functions in 2015, a fall from 17% (95% CI 7–39) in 2012.

### Proportions of women delivering in facilities ready to perform routine care signal functions

**Fig. 1:**
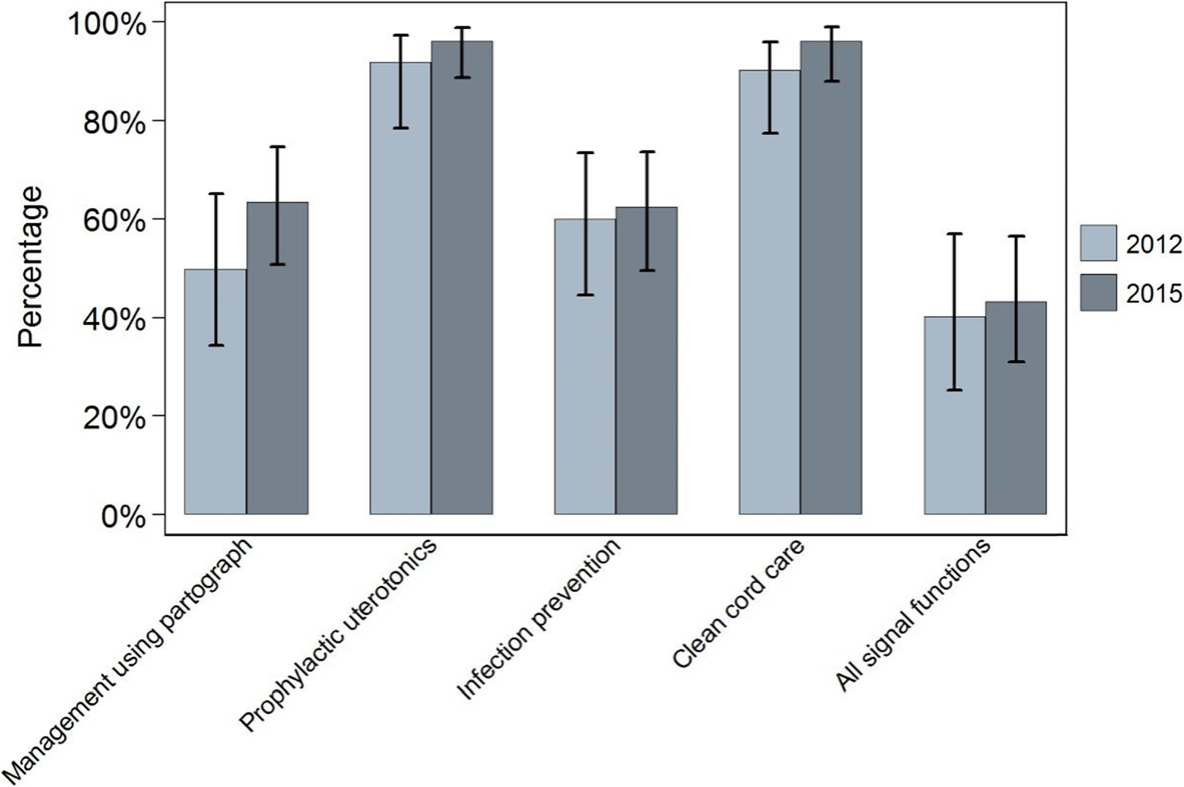
Ethiopia – proportion of all deliveries in facilities offering routine care signal functions

**Fig. 2:**
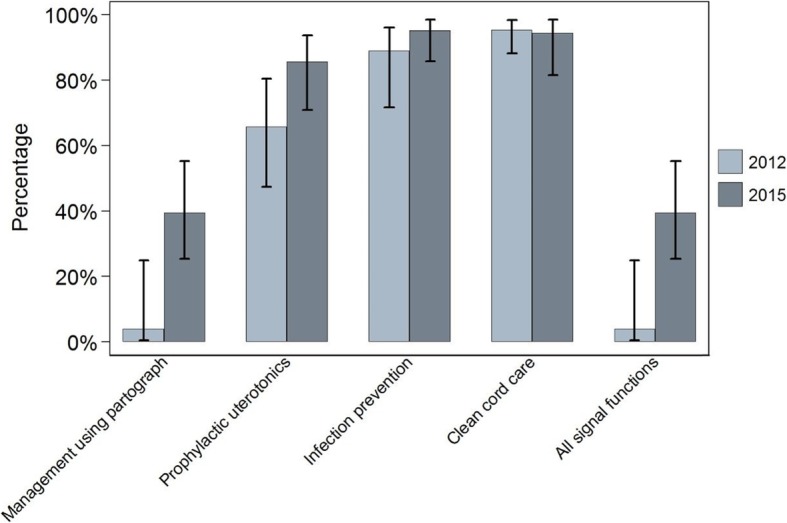
Uttar Pradesh – proportion of all deliveries in facilities offering routine care signal functions

**Fig. 3:**
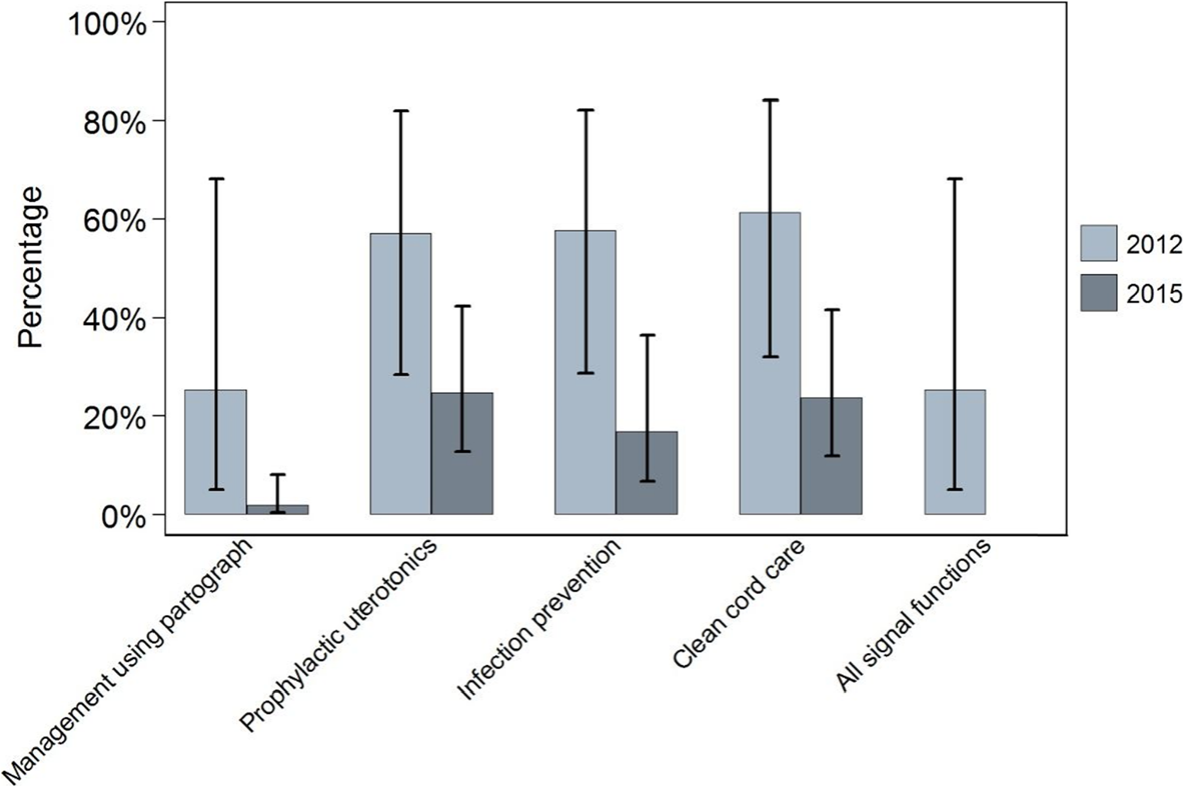
Gombe State – proportion of all deliveries in facilities offering routine care signal functions

Figures 1, 2 and 3 show, for each setting in 2012 and 2015, the proportion of all deliveries at sampled facilities in the six months prior to the surveys that took place in facilities ready to perform each signal function. In Ethiopia in 2012, 40% (95% CI 26–57) of primary facility deliveries in the six months prior to the survey took place in facilities that were ready to perform all four basic care signal functions at the time of the survey. This proportion was 43% (95% CI 31–56) in 2015. In Uttar Pradesh, the proportions of deliveries in primary facilities that were ready to perform all four signal functions were 4% (95% CI 0.6–24) in 2012 and 39% (95% CI 26–55) in 2015. In Gombe State, 25% (95% CI 6–66) of primary facility deliveries took place in facilities that were ready to perform all four signal functions, but by 2015 no women gave birth in a facility which was ready to perform all four functions at the time of the survey. Taken together, 64,649 deliveries were recorded in our three-country sample of primary care facilities in 2015, but just 23,425 (36%, 95% CI 27–47) occurred in facilities that could be considered ready to offer good quality routine maternal and newborn care.

## Discussion

This study of the quality of routine obstetric and newborn care in health facilities in three settings with high maternal mortality revealed that between 2012 and 2015 the proportion of facilities that were ready to offer such care showed some increase in Ethiopia, a more substantial increase in the Indian state of Uttar Pradesh, but a decrease in Gombe State in Nigeria. After incorporating the number of women delivering in these facilities we observed that across these settings no more than 4 out of every 10 women who delivered in a sampled facility in 2015 gave birth in a setting with the staffing, infrastructure and commodities needed to provide basic maternal and newborn care.

In Ethiopia and the state of Uttar Pradesh the increased readiness to provide basic care were not driven by a greater presence of SBAs, which was consistently high, but rather by improved availability of simple commodities such as partographs, urine testing kits and prophylactic uterotonics. In both settings this increased the proportion of facilities that were ready to perform all four of the signal functions used to measure routine maternal and newborn care. As a result, between 2012 and 2015 the proportion of facility births that took place in these more prepared facilities showed some increase in Ethiopia and a more substantial increase in Uttar Pradesh.

The facilities we surveyed in Ethiopia also experienced a substantial increase in the volume of deliveries between 2012 and 2015, reflecting government-led initiatives to promote facility births [23]. There was little increase in the median number of deliveries at sampled facilities between 2012 and 2015 in Uttar Pradesh, where a central government incentive to encourage facility births – in the form of a conditional cash-transfer scheme – had been in place since 2005 [24]. This initiative is thought to have led to significant increases in facility deliveries in public facilities across India between 2005 and 2010 [25] with one evaluation estimating that, in Uttar Pradesh, the proportion of such deliveries increased from 17 to 47% between 2005 and 2008 [26]. Our study suggests that by 2012 the impact of this intervention in Uttar Pradesh may have attenuated.

Despite these improvements in Ethiopia and Uttar Pradesh, by 2015 there remained a number of missed opportunities in these settings to provide routine but potentially life-saving care in childbirth. In Ethiopia in 2015 approximately 43% of women who gave birth in sampled facilities in the six months prior to survey did so in facilities that were ready to perform all four routine care signal functions. In Uttar Pradesh the equivalent proportion was 39%.

In Gombe State, the proportions of primary facilities that were ready to perform all four routine care signal functions declined from approximately 25% in 2012 to 0% in 2015. Part of this change was due to a steep decline in the proportion of facilities that had an SBA present on the day of the survey. Difficulties in recruiting and retaining skilled midwives in North-Eastern Nigeria [27] were exacerbated by repeated health worker strikes across the country [28]. In addition, North-Eastern Nigeria had been subject to severe political instability since 2009 and, while this primarily affected states to the north and east of Gombe, an estimated 900,000 people had been internally displaced across the region by January 2015 [29]. It is plausible that this had placed further pressure upon the recruitment and retention of skilled medical personnel, and also influenced the capability of pregnant women to seek a facility delivery.

Insufficient supplies of essential commodities contributed to reduced facility readiness in all three settings, a finding that reflects the inability of many primary facilities in resource-poor settings to provide the most essential care, and which has been reflected in other sub-Saharan African countries [30]. Such has been the scale of this problem with regard to commodities that in 2012 the UN Commission on Life-Saving Commodities for Women and Children (UNColSC) was established [31, 32]. This aimed to address problems in the supply of key commodities in maternal and newborn health across 50 of the poorest countries. While the UNColSC focussed attention on different commodities to those assessed in this study (with the exception of the uterotonic oxytocin), its report identified a complex set of barriers that would apply to a broader range of equipment and supplies. These included weak regulatory bodies, insufficient returns on manufacturing investment, breakdowns in distribution, low demand and incorrect prescription or use of medicines [31]. In 2015 an evaluation of progress towards the Commission’s goals in 12 countries (of which Ethiopia and Nigeria were two) found that an average of 40% of facilities faced stock-outs of commodities and that persistent “bottlenecks” in supplies existed at country level [32].

The life-saving potential of good quality care at the time of birth is recognised as having an important impact upon the desire of women to deliver in health facilities. In a study in northern Uganda, a fully supplied health facility was shown to be the most important enabling factor for women to choose a facility birth, ahead of increased access and improved treatment by health workers [33]. Absent or poorly functioning equipment can also cause facility staff to feel incapable of providing adequate care despite their best efforts, which can have a damaging impact upon staff morale [34].

Developing methods that can appropriately reflect the quality of care available to service users, and produce actionable evidence without adding burden to countries, is an urgent priority. In this analysis we measured, via the signal function methodology, the capability of health facilities to provide a good standard of routine maternal and newborn care, but we did not measure the actual care that was delivered in practice. This is a limitation, and the inclusion of data on SBA behaviours and processes could have been even more revealing. However, a considerable strength of this study was the application of readily available data on the quality of health facility care to the denominator of the number of deliveries taking place in those facilities, in order to estimate the proportion of women who had access to potentially life-saving care. This methodological approach responds to a call to action by the global measurement community [35–38], has the potential to be integrated within routine data systems, is relatively low cost and is highly temporal. The use of a composite measure provides a useful summary of facility capability, while breaking down the components of each one makes it easier for health facility programmes to identify where to act.

The evidence presented here is not at the population level, since it refers only to women delivering in government owned primary-care facilities. Furthermore, applying cross-sectional input data to births recorded in facility registers during the previous six months does assume that facility inputs were relatively static, which may have led to an over- or under-estimation of results (depending on stock-out patterns). But, since the same methods were applied at each time and setting, the comparison between levels remains valid and the need for action clearly identified.

## Conclusion

Primary facilities in all three settings had sub-optimal inputs available for basic maternal and newborn care. Incorporating the volume of births per facility revealed that the majority of women who delivered in a primary health facility did not find the routine care that they may have expected or hoped for. This simple and affordable method for measuring this dimension of quality can help to reveal where and what action is needed to realise the potential of the primary health system to save more maternal and newborn lives.

## Data Availability

The survey questionnaires and resulting pooled dataset which formed the basis of this study are available from the LSHTM data repository (Data Compass). The dataset and instruments for Ethiopia can be found at 10.17037/DATA.129; for Uttar Pradesh they are at 10.17037/DATA.113; and for Gombe State they are at 10.17037/DATA.131

## Abbreviations

SBA: Skilled Birth Attendant
MMR: Maternal Mortality Ratio
IQR: Inter-Quartile Range
CI: Confidence Interval
UNColSC: United Nations Commission on Life-Saving Commodities for Women and Children
